# Recalcitrant Fovea-Involving Macular Fold After Uneventful Epiretinal Membrane Surgery

**DOI:** 10.22336/rjo.2025.20

**Published:** 2025

**Authors:** Matteo Mario Carlà, Carlos Mateo

**Affiliations:** 1 Ophthalmology Department, “Fondazione Policlinico Universitario A. Gemelli, IRCCS”, Rome, Italy; 2 Ophthalmology Department, Catholic University “Sacro Cuore”, Rome, Italy; 3 Instituto de Microcirugía Ocular (IMO), Barcelona, Spain

**Keywords:** epiretinal membrane, macular fold, perfluorocarbon liquid, metamorphopsia, macular detachment, BCVA = Best-Corrected Visual Acuity, BSS = Balanced Saline Solution, ERM = Epiretinal Membrane, ILM = Internal Limiting Membrane, LE = Left Eye, OCT = Optical Coherence Tomography, PFCL = Perfluorocarbon Liquid, PPV = Pars Plana Vitrectomy, RD = Retinal Detachment, RE = Right Eye, RPE = Retinal Pigment Epithelium, RRD = Rhegmatogenous Retinal Detachment, SD-OCT = Spectral-Domain Optical Coherence Tomography, SRF = Subretinal Fluid

## Abstract

**Purpose:**

To describe a case of recalcitrant fovea-involving macular folds developing after uncomplicated epiretinal membrane (ERM) peeling and causing intractable metamorphopsia.

**Methods:**

Case report.

**Results:**

A 22-year-old man with a stage 3 ERM, visual acuity (VA) of 20/100, and a history of scleral buckle underwent pars plana vitrectomy (PPV) with internal limiting membrane (ILM) peeling. One week after surgery, VA dropped to 20/125 with worsened metamorphopsia. Ophthalmoscopy revealed a fovea-involving full-thickness macular fold, with photoreceptor outer segments in opposition. After 3 weeks of follow-up without improvement, PPV with induction of localized retinal detachment was performed, combined with retinal massage. Moreover, perfluorocarbon liquid (PFCL) was employed to stretch the retina. Nevertheless, the macular fold and metamorphopsia were unchanged, even one year after the first surgery.

**Discussion:**

We hypothesize that, due to the highly contracted ERM, the retina may have separated from the RPE during peeling and folded over in the first postoperative days. Concurrently, incorrect patient positioning under air tamponade might have contributed to the vertical orientation of the fold.

**Conclusion:**

Even if macular folds after ERM surgery are rare, prompt surgical treatment rather than watchful waiting should be considered to prevent permanent functional impairment.

## Introduction

Macular folds are reported as a rare consequence of pars plana vitrectomy (PPV) or scleral buckling with intraocular gas tamponade for retinal detachment (RD) [1]. Nevertheless, the actual prevalence of macular folds remains unclear, with recent research reporting a 2.8% rate of occurrence after retinal detachment (RD) repair with a scleral buckle [2]. The pathogenesis of macular folds is believed to depend on persistent subretinal fluid (SRF) that is trapped under the macula during gas tamponade. Consequently, when the remaining SRF pools and settles underneath the neuroepithelial tissue, redundant retina is created at the boundary of the gas bubble [2].

Successively, the retina continues to roll over itself when the SRF resorbs, resulting in one or more folds. Numerous risk factors have been identified: superior bullous RD, intraocular gas tamponade, massive buckles, insufficient internal drainage of SRF, external drainage of SRF, and an RD whose border passes through the fovea [3]. Conservative care is considered suitable for peripheral folds; in most instances, these folds resolve spontaneously [4]. Conversely, aggressive treatment is recommended for macular folds because outer retinal degeneration can occur even in the first post-operative week, and structural disruption tends to chromicize [4,5].

However, the development of macular folds after vitrectomy in the presence of an attached retina is scarce, with only a few reports previously described [6,7]. This case report describes a case of recalcitrant fovea-involving macular folds that developed after uncomplicated epiretinal membrane (ERM) peeling and caused intractable metamorphopsia. Informed consent was obtained before publication.

## Case description

**Fig. 1:**
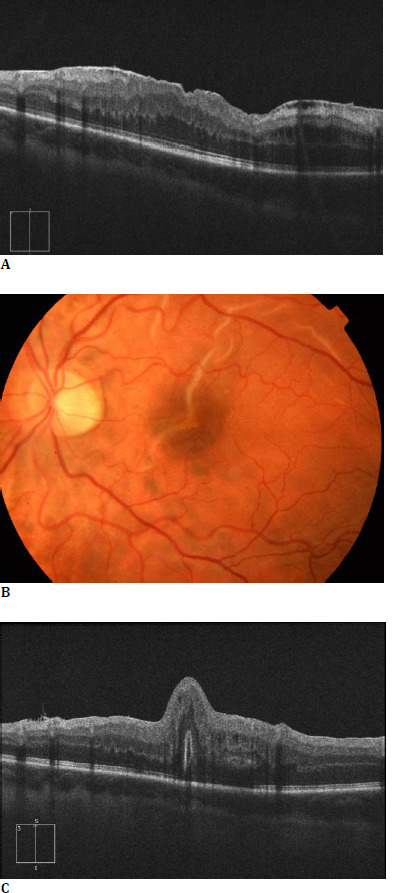
Collage of (**A**) preoperative OCT scan of the 22-year-old man showing a stage 3 ERM and post-operative fundus photography (**B**) and OCT (**C**) 1 week after surgery, underlining the presence of a vertically oriented full-thickness macular fold passing through the fovea. The visibility of photoreceptor outer segments in opposition, in the OCT scan, should be noted

**Fig. 2:**
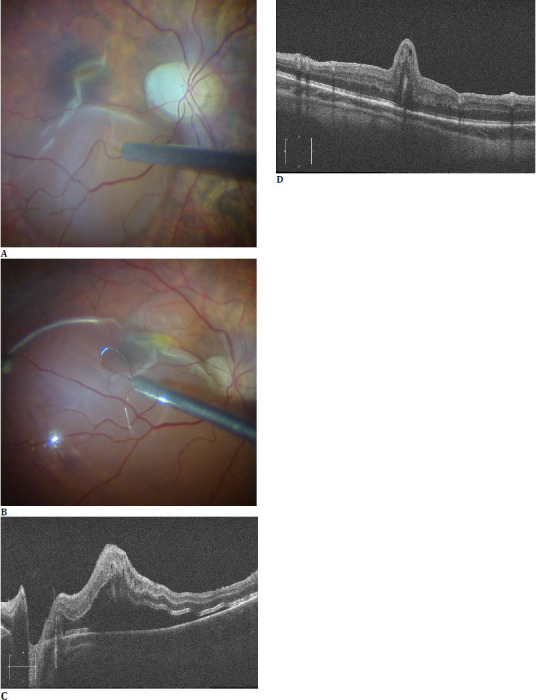
Screenshots from intraoperative recording highlighting the induced localized retinal detachment (**A**) performed with the 41-gauge needle and the gentle retinal massage (**B**) performed with the finesse flexloop. Post-operatively, OCT scans show gradual reabsorption of the subretinal fluid (**C**) but the unchanged macular fold, even one year after the first surgery (**D**)

A 22-year-old man was referred to our clinic at the Instituto de Microcirugia Ocular, complaining of great metamorphopsia and low vision in his left eye (LE). He had a history of RRD treated with scleral buckle two years before and no indications of any systemic disease. His best-corrected visual acuity (BCVA) at presentation was 20/20 in the right eye (RE) and 20/100 in the left eye (LE), with a positive Amsler grid test. During the dilated fundoscopy examination, the retina of the LE was attached entirely, and a horseshoe-shaped retinal break at 3 o’clock was correctly managed by the scleral buckle and surrounded by a cryopexy scar. Visible retinal wrinkling was identifiable in the macular area. Spectral-domain optical coherence tomography (SD-OCT) confirmed the presence of a stage 3 ERM with ectopic inner-foveal layers and retinal thickening but no SRF (**Fig. 1**). A 25G PPV (Dorc Eva Nexus, DORC) with peeling of the ERM and internal limiting membrane (ILM) was performed. At the end of the procedure, an air-fluid exchange was performed, and an air tamponade was left in the vitreous chamber. One week after surgery, the patient presented with headache, double vision, and worsened metamorphopsia, with the BCVA dropping to 20/125. Fundus examination and SD-OCT revealed the presence of a vertically oriented, fovea-involving full-thickness macular fold, with photoreceptor outer segments in opposition (**Fig. 2**). For the next three weeks, we decided to follow up the patient, hoping for spontaneous resolution of the fold.

Since no changes were noted one month after the first surgery, we decided on surgical treatment. A 23G PPV was performed: initially, brilliant blue G (0.025%) was injected to seek for possible ILM remnants. Successively, a 41G needle was employed to inject balanced saline solution (BSS) in the subretinal space, creating a localized retinal detachment starting from the superior temporal arcade and involving the macular area. At this time, we realized that the inferior portion of the macular fold was firmly attached to the underlying retinal pigment epithelium and very difficult to detach. After fluid-air exchange, the aspirating cannula and the Finesse Flex Loop (Alcon Laboratories, Inc., Fort Worth, TX) were used to gently massage the retinal surface, aiming to relieve tissue redundancy, but with unsatisfactory results. Moreover, perfluorocarbon liquid (PFCL) injection was performed to stretch the retina and dislodge the subretinal fluid (SRF), but the fold persisted unchanged. At the end of the surgery, SRF in the macular area was intentionally left, and the patient was instructed to maintain a supine position for 4 days to facilitate the gradual reabsorption of fluid through the RPE (**Fig. 2**).

Nevertheless, OCT scans revealed a persistently unchanged macular fold at successive follow-ups. BCVA was stable at 20/100, and the patient’s distortion symptoms remained the same, even one year after the first surgery.

## Discussion

Retinal folds are a rare complication of vitreoretinal surgery, typically occurring after the surgical correction of retinal detachment (RD). Retinal folds may partially impact a portion of the outer or inner retinal layers, or they can affect the full-thickness retina with photoreceptor outer segments in opposition. Discrete partial folds may sometimes be challenging to evaluate biomicroscopically, whereas full folds can often be observed quickly via clinical examination. Retinal folds usually resolve spontaneously over a prolonged follow-up period of several months; nevertheless, surgical revision may be necessary in cases of foveal involvement and associated symptoms, even if the specific surgical technique is not standardized [1].

Retinal folds following macular surgery with adherent retina are of even rarer occurrence. Iafe et al. reported a case of membrane peel-induced partial-thickness taco and ripple-shaped outer retinal folds after PPV, which resolved independently after four months [8]. Similarly, McDonald et al. reported a case of three macular folds developing after ERM peel and fluid-air exchange, causing significant metamorphopsia. During a 1-year follow-up, OCT revealed substantial improvement of the outer retinal layer folds, but symptoms did not improve [7]. In our case, the full-thickness macular fold involved the fovea. It caused significant visual distortion but showed no structural changes at the most recent OCT, even after the tentative surgical repair was performed.

Pathogenetically, a retinal fold suggests more surface area of the retina or superfluous retina due to the operation [9]. After RD repair, the retina may extend due to residual SRF, increasing its surface area and enabling folding. After ERM peeling, the process behind macular folding is more enigmatic. In previous reports of Iafe et al. and McDonald et al. [7,8], a history of retinal break was generally present, treated with laser retinopexy. In our case, the patient had undergone scleral buckle and retinopexy 2 years before, and no signs of chronic macular SRF were present. Therefore, it is unlikely that the SRF surrounding the retinal break tracked under the macula.

Due to a highly contracted and thickly adhered epiretinal membrane (ERM) in our patient, the retina may have been stretched during the development of the ERM. When the peeling was performed, it could separate from the RPE and fold over in the first post-operative days. Concurrently, incorrect patient positioning under air tamponade may have contributed to the vertical orientation of the fold in our case, compared with the classic horizontally oriented retinal folds reported in previous studies [10].

As a further complication, the optimal treatment for macular folds remains unknown. Wong suggests cautious therapy for patients with outer retinal folds; for full-thickness folds, surgery should be kept for the most severe instances [11]. Despite the surgical approach, which combined induced macular detachment, retinal massage, and PFCL-aided retinal stretching, the fold in our patient remained strongly recalcitrant and unsolved. However, waiting for spontaneous resolution has taken months from the first to the second surgery. Thus, we hypothesized that prompt treatment is advisable in full-thickness folds involving the fovea, as retinal duplication may exhibit loosened adherence in the first postoperative days, making it more susceptible to retinal massage or PFCL-aided stretching. In our case, the one-month elapsed time rendered the contact between the photoreceptor’s outer segments unbreakable, with no chance of improvement.

## Conclusion

In conclusion, the development of macular folds after ERM peeling vitrectomy is rare. However, when visual symptoms and OCT follow-ups highlight the presence of a fovea-involving fold, prompt surgical treatment rather than watchful waiting can offer a better visual prognosis.
